# Origin of the catalytic activity of phosphorus doped MoS_2_ for oxygen reduction reaction (ORR) in alkaline solution: a theoretical study

**DOI:** 10.1038/s41598-018-31354-0

**Published:** 2018-09-05

**Authors:** Cheng Liu, Huilong Dong, Yujin Ji, Tingjun Hou, Youyong Li

**Affiliations:** Institute of Functional Nano & Soft Materials (FUNSOM), Soochow University, Suzhou, Jiangsu 215123 China

## Abstract

Phosphorus doped MoS_2_ nanosheets (P-doped MoS_2_) have been reported as excellent oxygen reduction reaction (ORR) catalysts with four-electron selectivity in alkaline solution. By performing density functional theory (DFT) calculations, we revealed the detailed reaction mechanism and the key reaction sites on surface of P-doped MoS_2_ for ORR catalysis. The double P-doped MoS_2_ (2P-MoS_2_) is calculated to be more stable than the single P-doped MoS_2_ (P-MoS_2_), and the configuration with two P atoms in neighboring sites exhibits the highest stability. The surface of P-doped MoS_2_ is found highly active for dissociation of O_2_. Comparative calculations reveal that P-MoS_2_ is unsuitable as ORR catalyst due to the high dissociation barrier of H_2_O (1.19 and 2.06 eV for the first and second adsorbed H_2_O), while the 2P-MoS_2_ shows good ORR catalytic activity with much lower dissociation barrier of H_2_O (0.62 eV). Furthermore, we elucidated that the ORR catalytic activity in 2P-MoS_2_ originates from the activated S2 atom, which provides an extra adsorption site for the first H_2_O and the following OH group benefited from the enhanced hydrogen bond interaction. Our results illustrate the mechanisms of doped MoS_2_ based catalysts and provide rational way for designing ORR catalysts with high activity.

## Introduction

Because of the high energy density, high theoretical efficiency, and negligible emission of exhausted gases, fuel cells (FCs) are considered to be one of the most promising energy conversion devices^1,2^. As the crucial process in FCs, the oxygen reduction reaction (ORR) has been extensively studied^3–5^. Platinum (Pt) based ORR electro-catalysts are widely used because of their relatively low over-potential in practical applications^6–8^. However, due to their high cost, less abundance, poor stability in an electrochemical environment, and still sluggish kinetics^5,9,10^, there are worldwide research efforts to find precious-metal-free catalysts as replacement to Pt based catalysts, such as nonprecious metals, their alloys or oxides, and nitrogen-coordinated metals^11–13^. As the ideal candidates, metal-free carbon materials doped with heteroatoms (N, B, Si, P, S and F), have been demonstrated to be effective precious-metal-free ORR catalysts^14–25^.

Since the discovery of the ultrathin MoS_2_ monolayer, the electro-catalytic property has attracted great attention^26–29^. It was reported that with the decrease of monolayer size, MoS_2_ reveals favorable performance in ORR and hydrogen evolution reaction (HER)^29^. By examining nine different layered group 5 metallic transition-metal dichalcogenides (TMDs), Chia *et al*. identified VTe_2_ as the best electrocatalyst for the HER in terms of HER overpotential and Tafel slope, at 0.5 V vs. RHE and 55 mV dec^−1^, respectively^30^. Also, other TMDs, such as TaS_2_, have been found to be efficient catalysts for the ORR due to the onset potentials similar to that of Pt/C catalysts^31^. Luxa *et al*. held the view that the increased activity of 2HTaS_2_ may actually stem from the presence of oxides (Ta_2_O_5_) formed during the oxidation^31^. Another way to improve the electrocatalysis activity is to transform the TMDs from the semiconducting 2H phase to the metallic 1T phase, such as the improved HER performance on the catalyst MoS_2_ and WS_2_ with p-doping Niobium and Tantalum^32^. Both experiments and theoreticians suggested that the band gap of TMDs can be tuned by creating their solid solutions either by having metal dichalcogenides with mixed metal elements (i.e., Mo_x_W_1−x_S_2_ (0 ≤ x ≤ 1)^33^ or mixed chalcogenides (i.e., MoS_y_Se_2−y_ (0 ≤ y ≤ 2))^34^, which make a contribution to electrocatalytic properties toward HER^35^. In order to improve the performance in electro-catalysis without the using of precious metal, N- and P-doped MoS_2_ with few layers were prepared experimentally. The experimental data indicate that N- and P-doped single layered MoS_2_ possess wonderful ORR catalytic performance in acidic and alkaline environment, respectively^36,37^. Soon after, the mechanism about the ORR catalytic effect on N- and P-doped MoS_2_ single layer is studied by simulations^38^. However, the catalytic mechanism on the experimental results of P-doped 2D materials as ORR catalysts is not studied, especially the reported generation of extra ORR activity on P-doped MoS_2_ nanosheet with four-electron selectivity in alkaline solution, which have been successfully synthesized via the method of pyrolysis^37^. More importantly, Huang and the co-worker have found through the mapping analysis that, P doping on the MoS_2_ monolayer can be dispersed on the surface evenly, and with the increase of P-doping amount, the activity of P-doped MoS_2_ on ORR can be improved obviously, which reveals that the active site should be related with the P atoms.

In this work, by density functional theory (DFT) calculations, we explored the origin of catalytic activity in the P-doped MoS_2_ monolayer as the ORR electrocatalyst. Previous theoretical and experimental studies^37^ have indicated that, the P-doped MoS_2_ monolayer prefers to work as ORR catalyst in alkaline solution rather than acid solution. According to our simulation results, the P-doped MoS_2_ with doping concentration of 5.5% is optimal for ORR catalytic activity (the optimal value observed experimentally is 4.7%^37^), since it results in stable adsorption of H_2_O molecule and the extra OH group through the activated S site, and facilitates the following ORR steps to proceed with a lower barrier in alkaline media. Our work reveals the origin of catalytic activity of P-doped MoS_2_ in catalyzing ORR in alkaline solution and provides rational proposal for designing novel ORR catalysts.

## Results and Discussion

### Characterization of P-doped MoS_2_

Before the researches on ORR catalytic performance of P-doped MoS_2_ nanosheets, we first characterized their structural stability, structural and electronic properties, and pristine MoS_2_ 3 × 3 supercell is taken as comparison. The stability of pristine MoS_2_ and P-doped MoS_2_ are estimated by computing their formation energies (E_f_) as follows,

1 $${E}_{f}=({n}_{Mo}{\mu }_{Mo}+{n}_{s}{\mu }_{s}+{n}_{P}{\mu }_{P}-{E}_{total})/N,$$

where *E*_*total*_ is the total energy of the nanosheet, *n*_*Mo*_, *n*_*S*_, and *n*_*P*_ are the numbers of Mo, S, and P atoms in the cell. *N* is the total number of the atoms. *μ*_*Mo*_, *μ*_*S*_, and *μ*_*p*_ are the chemical potentials of Mo, S, and P, respectively. *μ*_*P*_ is obtained from black phosphorus, while *μ*_*Mo*_ is obtained from bulk molybdenum and *μ*_*S*_ is obtained from bulk MoS_2_ by subtracting the chemical potential of Mo, respectively. According to the definition, the formation energies of MoS_2_, P-MoS_2_ and three types of 2P-MoS_2_ are calculated as −0.052, −0.070, −0.103, −0.105, and −0.105 eV/atom, respectively (as listed in Table 1). Compared with the pristine MoS_2_ and P-MoS_2_, 2P-MoS_2_ monolayers exhibit very close formation energy (around 0.1 eV/atom), indicating that the P-doping concentration can be increased from 3.7 atom% to 5.5 atom% easily on pristine MoS_2_ monolayer.

**Table 1:** The calculated bond energy, the height of P atom and formation energy for pristine, P-MoS_2_ and 2P-MoS_2_.

	Mo-P bond energy (eV)	Relative height of P (Å)	Formation energy (eV/atom)
MoS_2_	–	0	−0.052
P-MoS_2_	4.847 (Mo-P)	−0.023	−0.070
2P-MoS_2_(1)	4.966 (Mo-P)	−0.109	−0.077
2P-MoS_2_(2)	4.846 (Mo-P)	−0.089	−0.079
2P-MoS_2_(3)	4.881 (Mo-P)	−0.093	−0.079

The bond energy is another parameter to be compared. The bond energy is calculated as followed:

2 $${{\rm{E}}}_{bond}=(n{E}_{P}+{E}_{D}-{E}_{total})/n,$$

where *E*_*total*_ is the total energy of nanosheet, *n* is the number of P atom, the *E*_*P*_ and *E*_*D*_ denote the energy of isolated P atom and the energy of MoS_2_ sheet with vacancy defect (VS), respectively. As listed in Table 1, all the 2P-doped ones have as twice as lager Mo-P bond energy than single P-doped one, indicating the significantly higher stability of Mo-P bond in 2P-MoS_2_ than that in P-MoS_2_. As a comparison, the bond energy of Mo-S bond in pristine MoS_2_ is 0.224 eV, which is only slightly larger than those of Mo-P bond in 2P-MoS_2_. Attributing to the neighboring position of P and S in periodic table, the doping of P doesn’t affect the initial structure of MoS_2_ monolayer much, which can be confirmed by the few relative height of P in doped MoS_2_.

**Figure 1:**
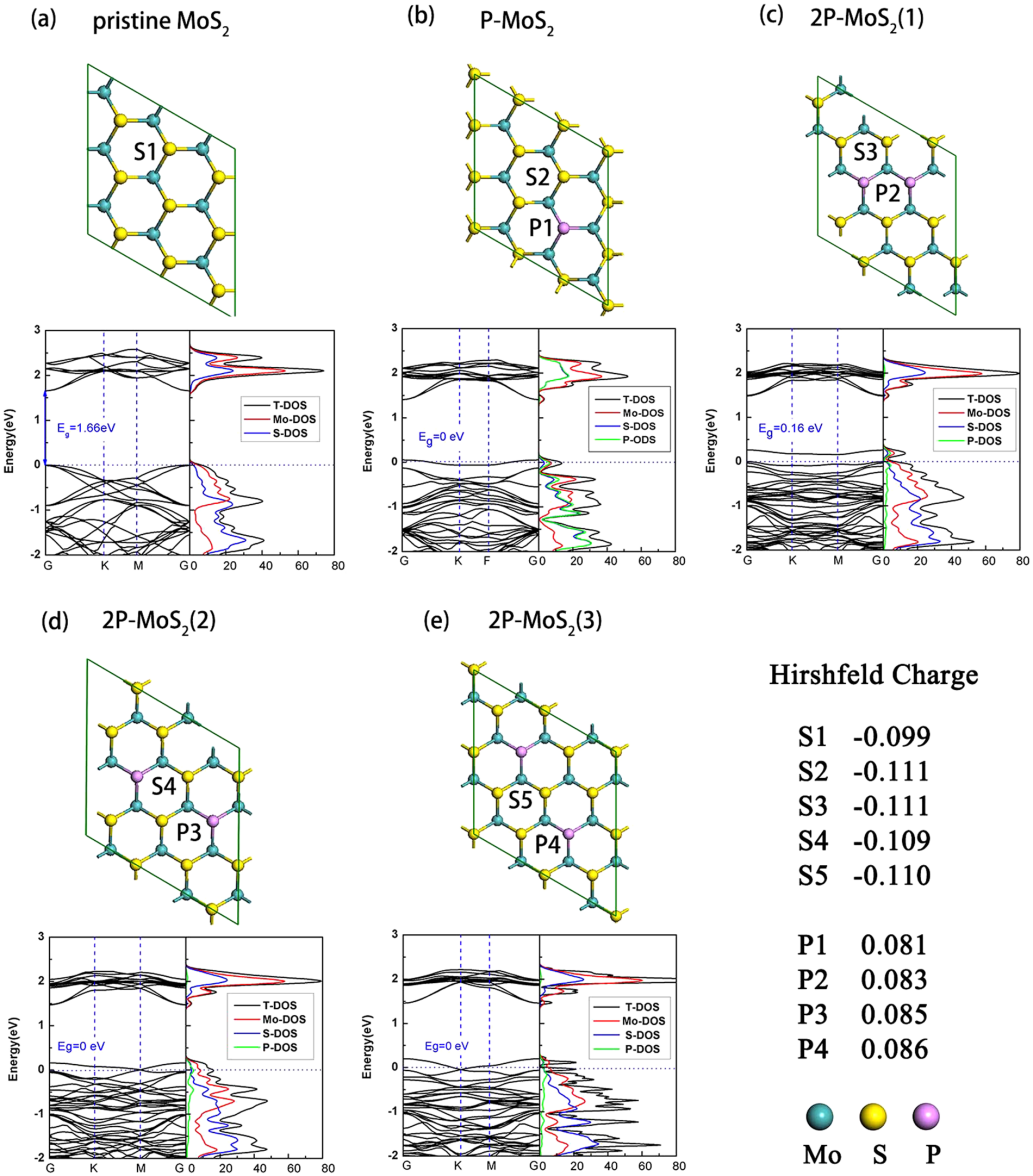
The top view of the optimized structure of (**a**) pristine MoS_2_ nanosheets, (**b**) single P-doped MoS_2_ nanosheets and (**c**–**e**) double P-doped MoS_2_ nanosheets, respectively. The band structures and corresponding partial density of states (PDOS) are also given. The Hirshfeld charge population of the corresponding atom is also given.

By comparing the internal energy of three different 2P-doping MoS_2_ monolayers through different softwares (see Table S1), we confirm that the doped P atoms in MoS_2_ tend to distribute locally (Fig. 1c) other than separately. Thus the first type of 2P-doping MoS_2_ should be the most stable configuration, which is the most possible to be experimentally prepared. This could be further validated by the tendency study. As shown in Fig. S3, we evaluated the tendency of increasing P doping concentration against the energy difference between locally distributed one and separately distributed one. A significant tendency is revealed that with the doping concentration of P increasing, the energy difference between locally distributed one and separately distributed one also increases. Thus it is not accidental that 2P-MoS_2_ (1) shows the highest stability among the three configurations, and P in P-doped MoS_2_ prefers to distribute locally.

Then the electronic properties of P-doped MoS_2_ are evaluated. As displayed in Fig. 1, we plotted the band structures of the P-MoS_2_ and 2P-MoS_2_, along with the pristine MoS_2_ as comparison. Our DFT results indicate that the doping of P leads to vanishing of energy gap [zero gap for P-MoS_2_, 2P-MoS_2_(2) and 2P-MoS_2_(3), and 0.16 eV for 2P-MoS_2_(1)] compared with the pristine MoS_2_ (1.66 eV). This is mostly due to the introduction of impurity band, which exists as a single band across or above the Fermi level that is splitting from valence band edge. Further analysis from partial density of states (PDOS) tells that the splitting of impurity band origins from the orbital hybridization between Mo and P, as shown from PDOS plots in Fig. 1. The small or zero gap induced by doping of P not only greatly enhances the conductivity of MoS_2_, but also facilitates the chemical reactivity.

Additionally, the difference of electronegativity between S and P atom also plays important role on the charge population. As shown in Fig. 1, the S atoms (S2–S5) surrounding the doped P atom carry more negative charge (about −0.11 |e|) than that in pristine MoS_2_ (S1, −0.099 |e|). Meanwhile P atoms carry positive charges (more than +0.08 |e|), which renders the P atom as suitable adsorption site for the nucleophilic species.

### The adsorption of O_2_ on P-doped MoS_2_

**Figure 2:**
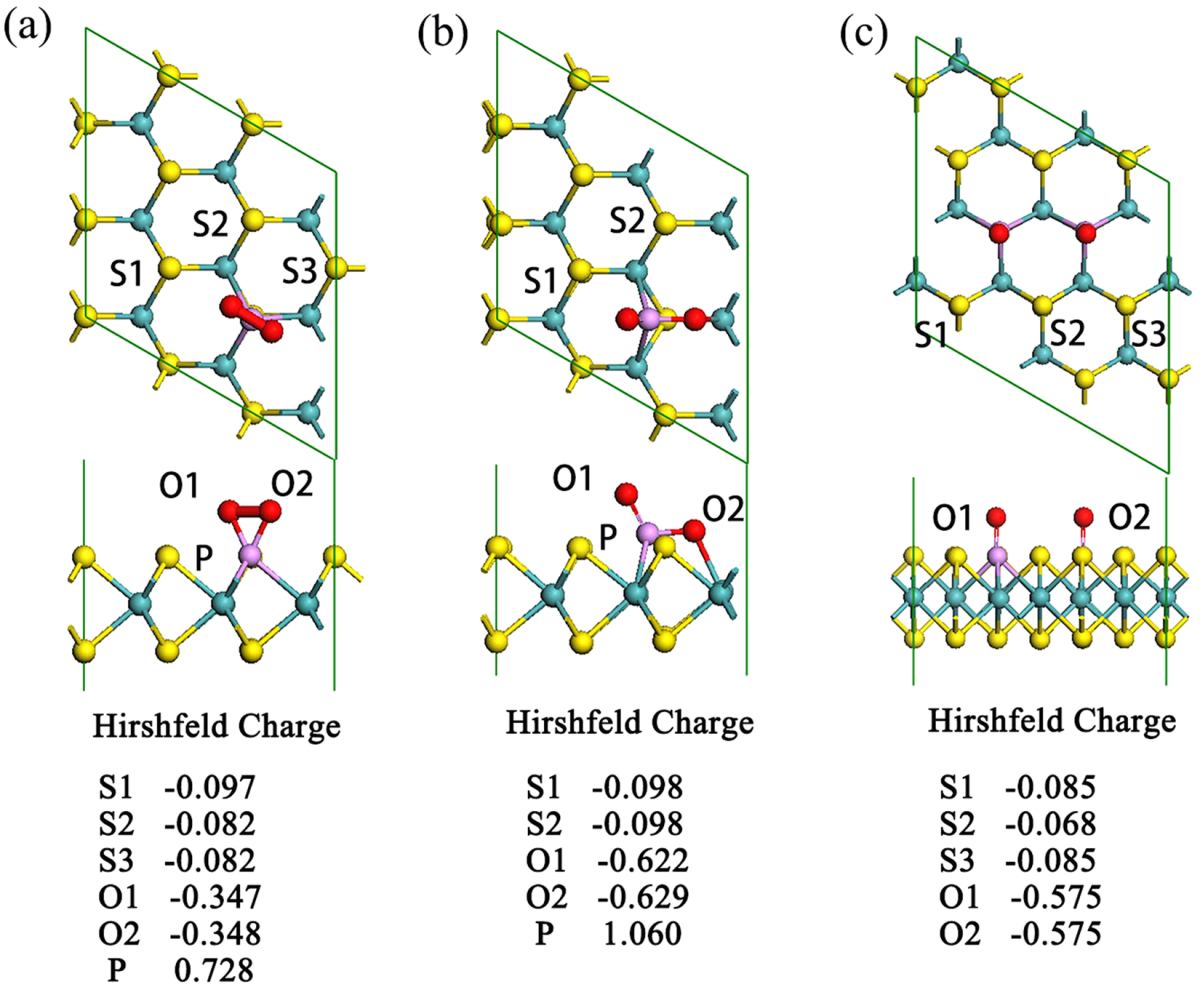
The top and side views of the optimized adsorption configurations of (**a**) O_2_ adsorbed P-MoS_2_, (**b**) 2O adsorbed P-MoS_2_ and (**c**) 2O adsorbed 2P-MoS_2_ (1). The Hirshfeld charge values of the corresponding atoms are also given.

As the very beginning of ORR, adsorption of O_2_ is taken into consideration first. By putting O_2_ molecule on different sites on P-MoS_2_ and comparing their E_ads_, we determined the most energetically favorable adsorption configuration as shown in Fig. 2a. As we can see, the O_2_ molecule chemisorbs to the P-MoS_2_ by bonding with P atom. The bond length of P-O bonds is about 1.670 Å. And the calculated adsorption energy of O_2_ on P-MoS_2_ is up to E_ads_ = −1.11 eV, which is much more favorable than that on pristine MoS_2_ monolayer (−0.11 eV). And the charge transferred from P-MoS_2_ to O_2_ is as much as Q = 0.695 |e| (in ref.^33^, E_ads_ = −0.93 eV and Q = 0.30 |e|). Further results from TS search show that the chemisorbed O_2_ can be easily dissociated into two O atoms by overcoming a negligible energy barrier (about 0.05 eV). As shown in Fig. 2b, the two dissociated O atoms adsorb on the top site of P atom and the bridge site between P and Mo atoms, respectively. Similarly, the adsorption of other ORR species (OH and H_2_O) also prefers to adsorb onto the P atom, as displayed in Fig. S1 of Supporting Information.

There are two different occasions for adsorption of O_2_ molecule on 2P-MoS_2_. For 2P-MoS_2_ (1), the O_2_ molecule binds with the two neighboring P sites with bridged configuration. For 2P-MoS_2_ (2) and (3), it is found that due to the large distance between the two P atoms, O_2_ molecule can only separately bind with one P atom as the situation of P-MoS_2_ (Fig. S1). In the following discussion, we only investigated the adsorption performance of O_2_ on 2P-MoS_2_ (1), not only due to its highest stability among the 2P-MoS_2_, but also for its different binding configuration with O_2_ compared with P-MoS_2_. Resulting from the high chemical activity of the two adjacent P atoms, the O_2_ molecule will directly dissociated and form two P=O bonds without any energy barrier, as shown in Fig. 2c. The bond length of the two P=O bonds are 1.505 Å, which is shorter than the P=O bond (1.515 Å) formed on P-MoS_2_.

The charge population of P-MoS_2_ and 2P-MoS_2_ (1) after dissociation of O_2_ is also compared. It is found that comparing with the S atoms (S1 and S2) in P-MoS_2_, the S atoms surrounding dopants (S1, S2, and S3 in Fig. 2c) on 2P-MoS_2_ carry less negative electron, which may be favorable for the ORR steps as we will discuss in the following parts.

### ORR on P-MoS_2_

Generally, the total reaction in alkaline solution can be expressed as a 4-electron evolution (*denotes the adsorbed surface):

3 $${{\rm{O}}}_{2}(aq)+2{H}_{2}O+4{e}^{-}=4O{H}^{-}$$

The elementary reaction steps could be expressed as:

4 $${{{\rm{O}}}_{2}}^{\ast }\to 2{{\rm{O}}}^{\ast }$$

5 $$2{{\rm{O}}}^{\ast }+{{\rm{H}}}_{2}{\rm{O}}+2{{\rm{e}}}^{-}\to 2{{\rm{OH}}}^{-}+{{\rm{O}}}^{\ast }$$

6 $${{\rm{O}}}^{\ast }+{{\rm{H}}}_{2}{\rm{O}}+2{{\rm{e}}}^{-}\to 2{{\rm{OH}}}^{-}$$

**Figure 3:**
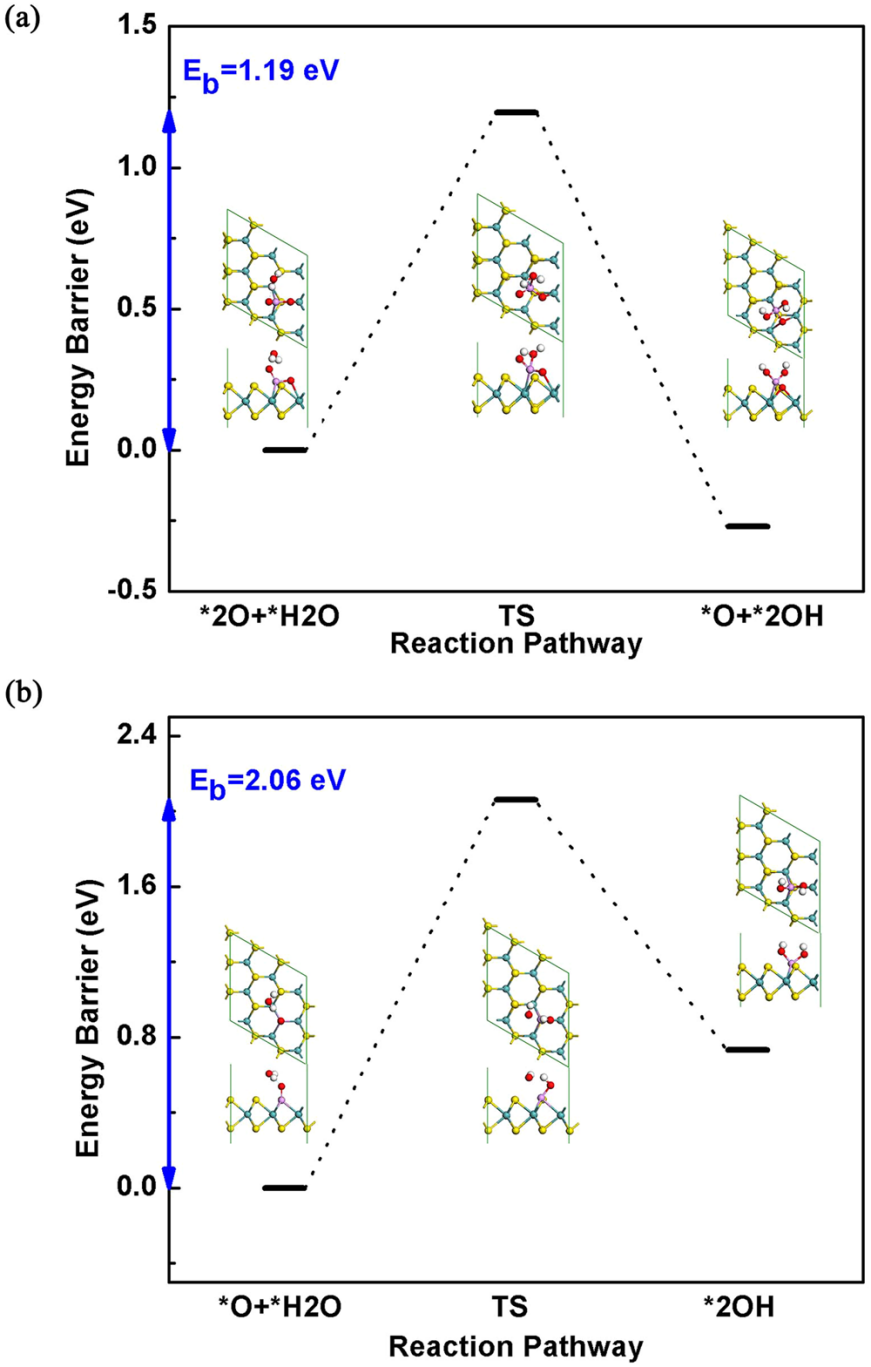
The top and side views of optimized reactant and product as well as the reaction energy barrier for the dissociation of (**a**) first H_2_O and (**b**) second H_2_O. E_b_ in the diagram represents the barrier energy for each step. The dash line between O atom and H in H_2_O represents the effect of hydrogen bonding.

After the determination of adsorption and dissociation of O_2_, we then proposed the ORR pathway in alkaline environment by taking H_2_O molecule into consideration. Due to the lack of adsorption site, our simulation results indicate that the first H_2_O can only adsorb on the top of P=O bond by hydrogen bond, as shown in Fig. 3a. One of the H atoms in H_2_O will then transfer to the P=O and finally form two adsorbed OH groups as the product. In the most stable configuration of (2OH* + O*), the bridged O atom will move to form two Mo–O bonds with the breaking of Mo–P bond, as a result of steric repulsion from the two OH groups. The activation barrier for this reaction is 1.19 eV, fairly high for room temperature reaction.

Similarly, the dissociation of second H_2_O molecule also takes place on the top site of the P=O bond, as shown in Fig. 3b. The activation barrier for this reaction is up to 2.06 eV, even higher than the dissociation of first H_2_O. Moreover, this reaction is endothermic with reaction energy of 0.73 eV, which is thermodynamically unstable. The high activation barrier for dissociation of H_2_O demonstrates that the catalytic activity of P-MoS_2_ is low for ORR, which mainly due to the strong P=O bond and low reaction activity of H_2_O on P-MoS_2_. This is in consistent with the previous theoretical investigations^38^.

### ORR on 2P-MoS_2_

**Figure 4:**
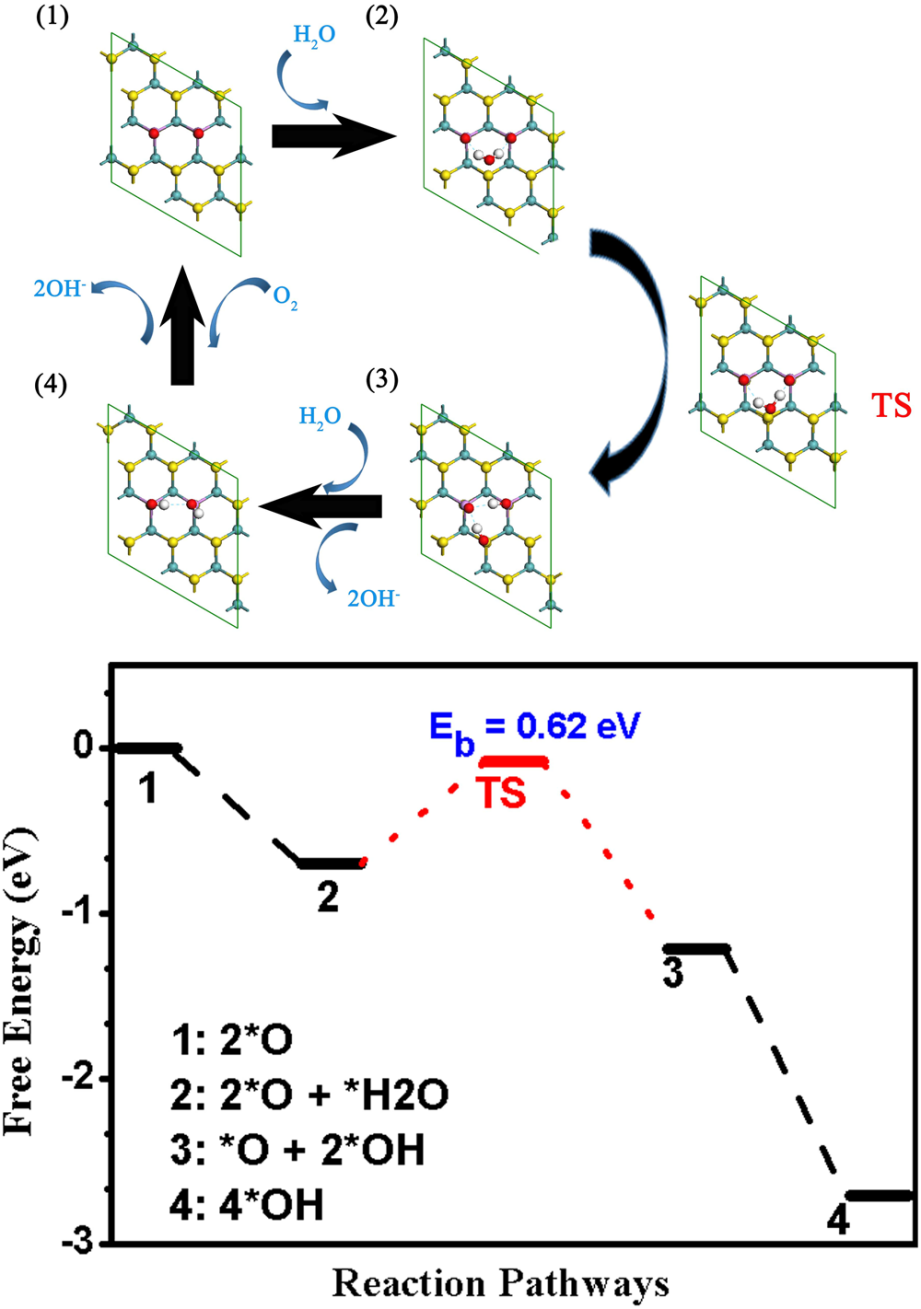
The optimized structure configuration of reactant, intermediates and product (1~4) and the corresponding reaction pathways of ORR on 2P-MoS_2_ sheet in alkaline environment.

As is presumed from the charge population and electrostatic potential (ESP, shown in Fig. S2) distribution, the two adjacent doping P atoms on the 2P-MoS_2_ (1) should be the most active sites for ORR. Same with the P-MoS_2_, H_2_O molecule physisorbs on 2P-MoS_2_, mostly relying on the hydrogen bonds between H atoms in H_2_O and P=O bonds, as shown in Fig. 4c. However, the adsorption of H_2_O on 2O adsorbed 2P-MoS_2_ (1) is obviously strengthened as listed in Table 2. It is found that the decrease of charge assigned on S2 (Fig. 2c) accounts for the improved adsorption performance of H_2_O. As we can see in Fig. 2c, due to the strong electronegativity of O, the presence of dissociated O atoms will decrease the negative charge of the surface S atoms. Interestingly, the S2 in 2O adsorbed 2P-MoS_2_ (1) exhibits much less negative charge (−0.068 |e| as marked in Fig. 2c) other than that in 2 O adsorbed P-MoS_2_ (−0.098 |e| as marked in Fig. 2b). It is the charge decrease as well as stronger hydrogen bond caused by the two nearby P–O bonds [as depicted in structure (2) of Fig. 4] together enhance the adsorption performance of H_2_O on the S2 site. Combined with the fact that the adsorption of H_2_O is strengthened, we assumed that it is this S2 atom that makes 2P-MoS_2_ (1) different, and further simulations confirm our proposal. We found that this S2 site can also serve as an adsorption site for OH group, which makes it active during the reaction. As the structure (2) displayed in Fig. 4, accompanied with the hydrogen bond interaction from two P=O bonds, the first added H_2_O molecule could be easily dissociated into two adsorbed OH groups, one binds with the P and the other one adsorbs onto the S2 site. The corresponding activation barrier for the dissociation of first H_2_O is 0.62 eV, a moderate value for reaction under room temperature. As comparison, both the two dissociated OH groups in P-MoS_2_ can only adsorb onto the P atom, leading to significant structural distortion and a high barrier of 1.19 eV.

**Table 2:** The adsorption energy of H_2_O molecule on the substrates of pristine MoS_2_, P-MoS_2_, 2P-MoS_2_, single O atom adsorbed P-MoS_2_ and 2P-MoS_2_, as well as two O atoms adsorbed P-MoS_2_ and 2P-MoS_2_, respectively.

	MoS_2_	P-MoS_2_	O- P-MoS_2_	2O- P-MoS_2_	2P-MoS_2_	O- 2P-MoS_2_	2O- 2P-MoS_2_
E_(ads)_/eV	−0.151	−0.129	−0.305	−0.256	−0.120	−0.372	−0.319

Following that, the formed OH groups would combine with the electron provided from the electrode and solve into the solution as OH−. And then, the exposed P atom will be the most active site on the surface of 2P-MoS_2_ (1). Both of the strong reaction activity of P atom and the negative charge on O atom contribute to the dissociation of the second H_2_O without energy barrier. Also, the possibility of the breaking of P=O bond and the formation of OH on P atom is strongly related with the assist of the hydrogen-bond. The whole reaction circulation and the corresponding energy profile of reaction pathway are shown in Fig. 4.

### Comparison between P-MoS_2_ & 2P-MoS_2_

Comparing 2P-MoS_2_ with P-MoS_2_, the most significant difference is the activation of the S nearby the doping P atoms (as shown in Fig. 2c) and the strong hydrogen-bond existed in 2O-2P-MoS_2_, which leads to the enhanced adsorption for H_2_O and the chemisorption of OH on S atom during the dissociation of H_2_O molecule. Resulting from the more stable adsorption of H_2_O as well as the OH group on the activated S site, the reaction energy barrier for the dissociation of the first H_2_O is greatly lowered compared with the P-MoS_2_ (0.62 eV for 2P-MoS_2_ and 1.19 eV for P-MoS_2_). Associated with the fact that the formation energy for 2P-MoS_2_ is almost the same with the P-MoS_2_ (Table 1), it is much easily for 2P-MoS_2_ to be prepared experimentally. Meanwhile, among the 2P-MoS_2_ nanosheets, type 1 is the most energetically favorable (Table S1). Based on the comparison results, we confirm that the activated S atom in 2P-MoS_2_ is the origin of ORR catalytic activity of P-doped MoS_2_ in alkaline solution. Notably, it is not accident that the 2P-MoS_2_ we modeled shows improved ORR catalytic performance. As we have stated, the doping concentration of 2P-MoS_2_ is 5.5%, which is quite close to the optimal doping concentration observed experimentally (4.7%)^37^. It is the special structure of 2P-MoS_2_ that activates the S2 site and facilitates the adsorption of H_2_O molecule and the extra OH group, which well explains why the MoS_2_ with a P doping concentration of 4.7% exhibits the best ORR performance in experiments.

## Conclusions

In summary, we performed comprehensive studies on the origin of the ORR catalytic activity of P-doped MoS_2_ in alkaline solution through DFT calculations. The 2P-MoS_2_ with doping concentration of 5.5% is calculated to be more stable than the P-MoS_2_ with doping concentration of 3.7%, which is consistent with the optimal doping concentration of 4.7% from the experiments^37^. And the configuration with two P atoms in neighboring sites is found to possess the highest stability among the possible configurations of 2P-MoS_2_. The surface of P-doped MoS_2_ exhibits highly reactive activation for dissociation of O_2_.

Except the doping on MoS_2_ plane, we also discuss the influence of phosphorus present on edge of MoS_2_. The detailed structure configuration and energy values have been given in Fig. S6. For the two edge S sites that can be placed by P (Mo edge and S edge as shown in Fig. S6 (a)), the P prefer doping in S edge site rather than Mo edge site considering the relative energy difference up to 0.97 eV. For the O_2_ adsorption on the P-doped MoS_2_ on the edge site, two adsorption sites are considered (shown in Fig. S6 (b)). The positive adsorption energy on the S site indicated that P-doped on the edge site can’t activate the S site around P atom. While on the edge P site, the adsorbed O_2_ would dissociated directly, which is same with single P atom doped on MoS_2_ plane (Fig. 2 (b)). And the adsorption for H_2_O is −0.13 eV, which is also the same with the P-MoS_2_ (−0.129 eV, as shown in Table 2). Thus, based on our calculation results, we concluded that the doping of P in the basal plane and edge sites will exhibit similar adsorption performance for adsorption of H_2_O and dissociation for O_2_.

Further comparative calculations reveal that P-MoS_2_ is unsuitable as ORR catalyst due to the strong P=O bond and high dissociation barrier of H_2_O on P-MoS_2_ (1.19 eV and 2.06 eV for the first and second adsorbed H_2_O). In comparison, the 2P-MoS_2_ shows good ORR catalytic activity. The dissociation barrier of the first H_2_O on 2P-MoS_2_ (0.62 eV) is much lower than that of P-MoS_2_. And the dissociation of O_2_ and the second H_2_O on 2P-MoS_2_ is even spontaneous without any activation barriers. While for the two other types of 2P-doped MoS_2_, not only the stability is less than the neighboring doped 2P-MoS_2_, but also the reaction mechanism is similar with the single P doped MoS_2_. By checking the charge population and adsorption performance of the ORR intermediates, we elucidated that the ORR catalytic activity in 2P-MoS_2_ originates from the activated S2 atom with increased charge that affected by the two neighboring P=O bonds, which provides an extra adsorption site for the first H_2_O and the following OH group with the help from the enhanced hydrogen bond interaction. Our results reveal the detailed reaction mechanism of MoS_2_ based catalysts and provides instructive suggestions for designing ORR catalysts with high performance.

## Methods

All the calculations are carried out by DMol^3^ program. The electronic exchange and correlation effects are described by the generalized gradient approximation with Perdew–Burke-Ernzerhof (GGA-PBE) functional^39^. In order to accurately describe the van der Waals forces, dispersion correction (DFT-D) is adopted by Grimme approach^40^. The all-electron double numerical atomic orbital including polarized p-function (DNP)^41^ is chosen as the basis set with orbital cutoff of 4.9 Å. The convergence threshold values for energies, gradient and displacement are specified as 1 × 10^−5^ Ha, 1 × 10^−3^ Ha/Å, and 5 × 10^−3^ Å, respectively, while the self-consistent-field (SCF) convergence threshold value is 1 × 10^−6^ Ha. To enhance SCF convergence efficiency during optimization, an electron thermal smearing value of 0.002 Ha is employed for all the calculations. The conductor-like screening model (COSMO)^42^ is used to simulate the aqueous environment, where the dielectric constant is set as 78.54 (water). All the stably existed species during the ORR process are confirmed no imaginary frequencies by performing frequency analysis. To determine the activation barriers (E_b_), transition state (TS) searches are conducted by using the complete linear synchronous transit/quadratic synchronous transit (LST/QST) method. All the obtained TS structures exhibit only one imaginary frequency throughout the potential surface, and are ensured to directly connect corresponding reactants and products by nudgedelastic band (NEB) algorithm^43^.

The unit cell of 2H-MoS_2_ is optimized by using 9 × 9 × 1 Monkhorst-Pack k-point grids. The single P doped MoS_2_ is then obtained by substituting a surface S atom in MoS_2_ 3 × 3 supercell into P atom, with a doping concentration of 3.7 atom%. The structures of MoS_2_ 3 × 3 supercell and single P-doped MoS_2_ nanosheet (P-MoS_2_) are depicted in Fig. 1(a,b). The double P-doped MoS_2_ nanosheet (2P-MoS_2_) is obtained by substituting two surface S atoms in MoS_2_ 2√3 × 2√3 supercell into P atoms, with a doping concentration of 5.5 atom%. Differing from the relative positions between the two doped P atoms, there are three possible configurations that are taken into consideration. That is, neighboring sites (type 1), spaced by a Mo atom (type 2), and spaced by a S atom (type 3), as shown in Fig. 1(c–e). Considering the close atomic volume between P and S atom, the influence of doping on lattice size is neglected. Brillouin zone of MoS_2_ 3 × 3 supercell and the P-doped MoS_2_ structures are sampled by 3 × 3 × 1 Monkhorst-Pack k-point grids during the geometry optimization, which is tested converging for total energy. Population analysis of electron is performed by assigning Hirshfeld charges^44^ for the optimized structures.

In this article, the adsorption energy (E_ads_) is defined as

7 $${E}_{ads}={E}_{mol/sub}-{E}_{mol}-{E}_{sub}$$

Where E_mol_ and E_sub_ represent the total energies of the isolated adsorbate and adsorption substrate, and Emol/sub represents the total energy of the adsorption system. Here, the negative Eads means exothermic process for adsorption. The more negative Eads is, the more stable the adsorption system is. The triplet is taken as the ground state of O2 during the calculation.

In order to calculate the Gibbs free energy of reactions involving electron/proton transfer, the computational hydrogen electrode (CHE) model introduced by Nørskov and co-workers was used^45^. The reaction free energies (ΔG) can be computed as follows:

8 $${\Delta }G={\Delta }E+{\Delta }ZPE-T{\Delta }S+{\Delta }{G}_{pH}+{\Delta }{G}_{U}$$

where the ΔE was the reaction total energy directly obtained from Dmol3 calculation, ΔZPE is the change in the zero-point energy, T is the temperature (298.15 K), and ΔS is the change in entropy. ΔG_pH_ = K_b_T × ln10 × pH represents the free energy contribution due to the variation in the H concentration, and the pH value in this work was assume to 14 in the alkaline medium. ΔG_U_ = −neU, where n was the number of electrons transferred and U was the applied electrode potential. The entropy values are taken from the physical chemistry table considering H_2_ and H_2_O in gaseous form at room temperature and atmospheric pressure whereas the entropy of the adsorbed state is considered to be negligible. ZPE of the free molecules are estimated from our DFT calculations considering vibrational frequencies of the molecules in the harmonic approximation. The details of calculation steps to plot Free energy profile are demonstrated in the supporting information.

## Electronic supplementary material

Supporting Information
