# Supplementary material for: Origin of the catalytic activity of phosphorus doped MoS2 for oxygen reduction reaction (ORR) in alkaline solution: a theoretical study
Source: Sci Rep. 2018 Sep 5;8:13292. doi: 10.1038/s41598-018-31354-0 (PMC6125367; doi:10.1038/s41598-018-31354-0)
Supplement: Supplementary file 1 — Supporting Information [file 41598_2018_31354_MOESM1_ESM.docx]

**Origin of the catalytic activity of phosphorus doped MoS_2_ for oxygen reduction reaction (ORR) in alkaline solution: a theoretical study**

Cheng Liu, Huilong Dong,* Yujin Ji, Tingjun Hou, and Youyong Li*

Institute of Functional Nano & Soft Materials (FUNSOM), Soochow University, Suzhou, Jiangsu 215123, China.

* Corresponding authors.

E-mail: huilong_dong@126.com, Tel: (86)-512-65882037;

E-mail: yyli@suda.edu.cn, Tel: (86)-512-65882037.

**Supporting Information**


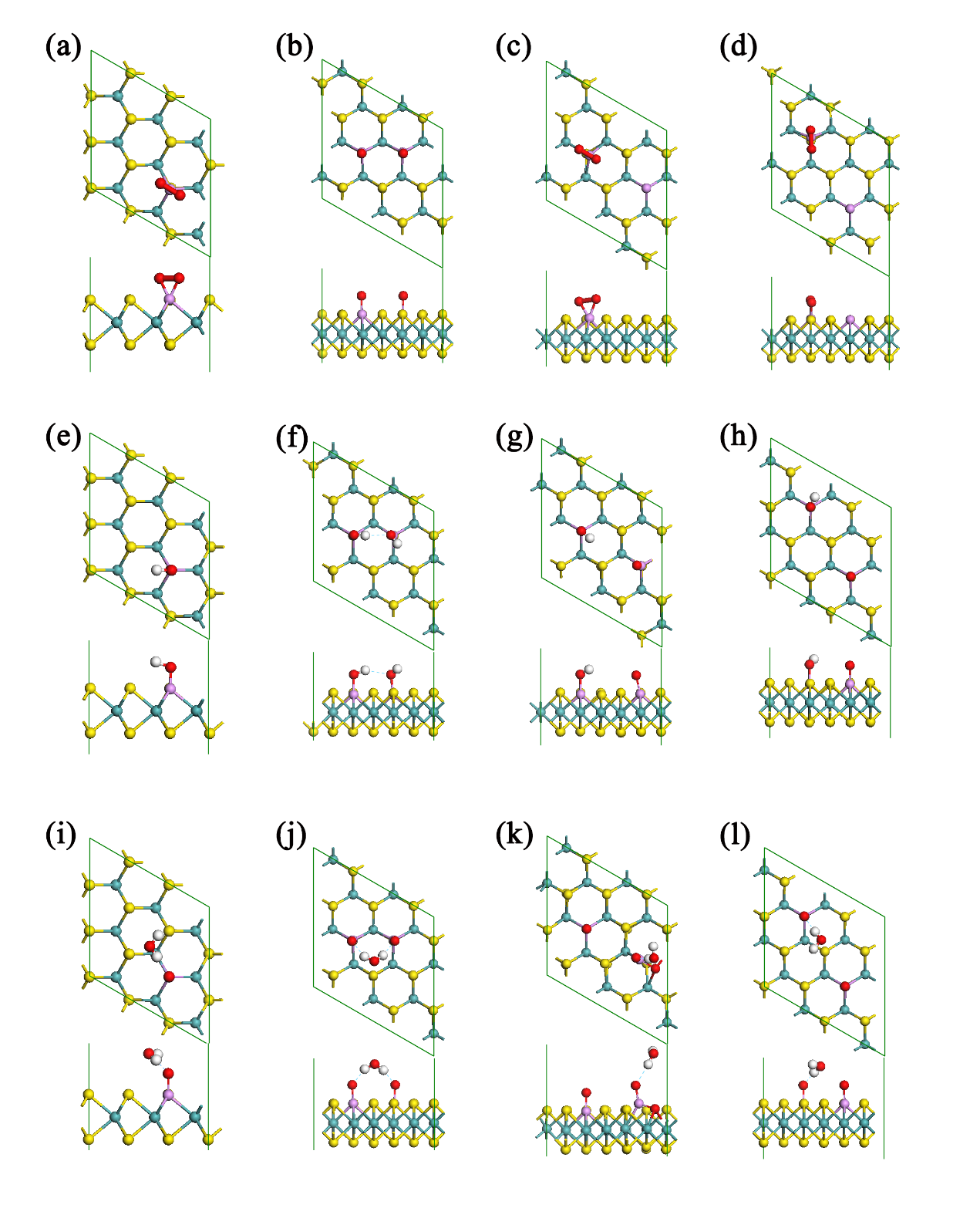


**Figure S1.** The stable configuration with the adsorption of O_2_, OH and H_2_O molecules for (a, e, i) P-MoS_2_, (b, f, g) 2P-MoS_2_ (1), (c, g, k) 2P-MoS_2_ (2), and (d, h, l) 2P-MoS_2_ (3), respectively.


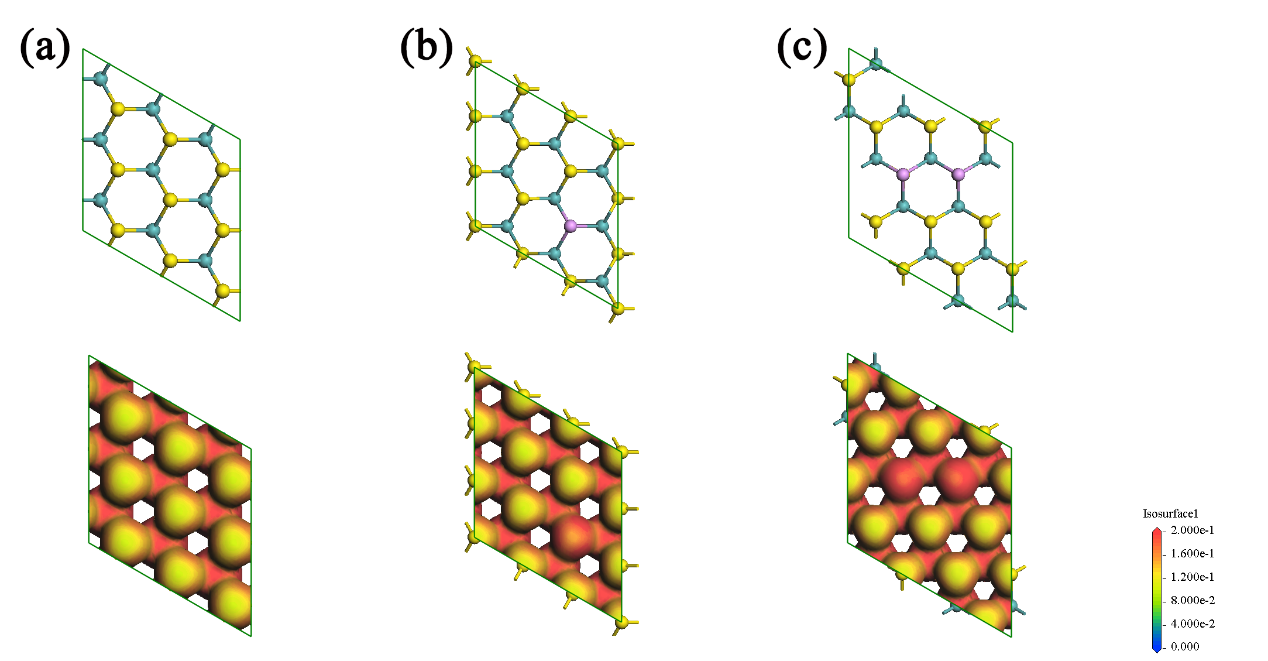


**Figure S2.** The electrostatic potential (ESP) analysis of (a) pristine MoS_2_, (b) P-MoS_2_ and 2P-MoS_2_.

**Table S1.** The differential energy of three different 2P-doped Mos_2_ monolayers simulated by Dmol^3^ and VASP, respectively.

|  | Differential Energy (E_dif_ /eV) | |
| --- | --- | --- |
|  | Dmol^3^ | VASP |
| 2P-MoS_2_-1 | 0 | 0 |
| 2P-MoS_2_-2 | 0.076 | 0.082 |
| 2P-MoS_2_-3 | 0.056 | 0.044 |

The differential energy of different 2P-doped MoS_2_ monolayers is obtained by taking the 2P-MoS2-1 as the energy reference. The calculation results from Vienna *ab initio* simulation package (VASP) is taken as comparison, with the setting of GGA-PBE functional and cut-off energy of 500 eV. By comparing the calculation results of Dmol^3^ and VASP, it is clear that both the results reveal that first type of 2P-doped MoS_2_ [2P-MoS_2_ (1)] should be the most stable among the three different 2P-doping configurations.


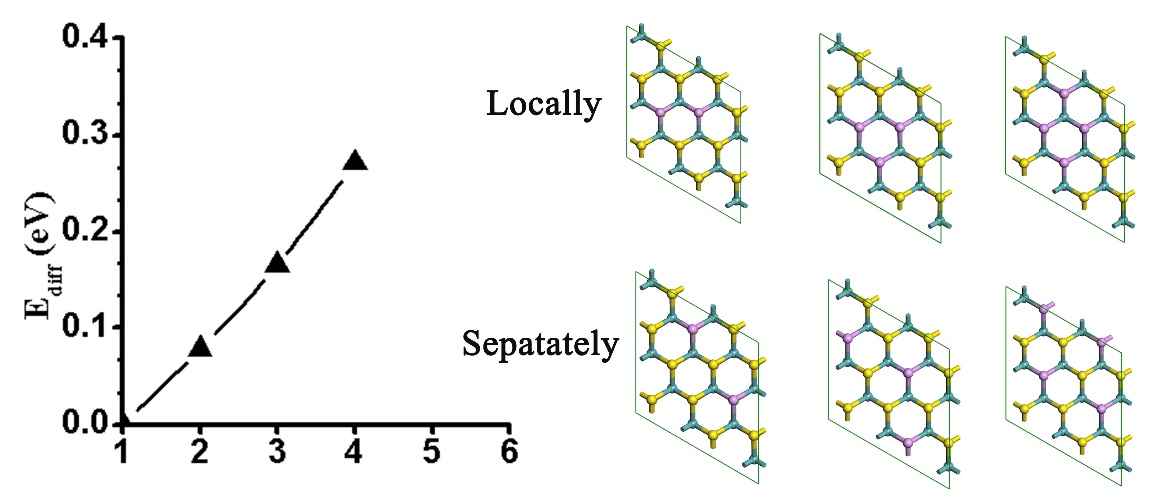


**Figure S3.** The curve of differential internal energy and the configuration of different doping states.

The differential internal energy is calculated as followed.

$E_{diff}=E_{\mathrm{separately}}- E_{locally}$

**Table S2.** The differential internal energy of three different P doping concentrate MoS_2_ monolayers obtained by Dmol^3^.

| Doping P amount | Differential internal energy (ev) |
| --- | --- |
| 2 | 0.0780 |
| 3 | 0.166 |
| 4 | 0.271 |


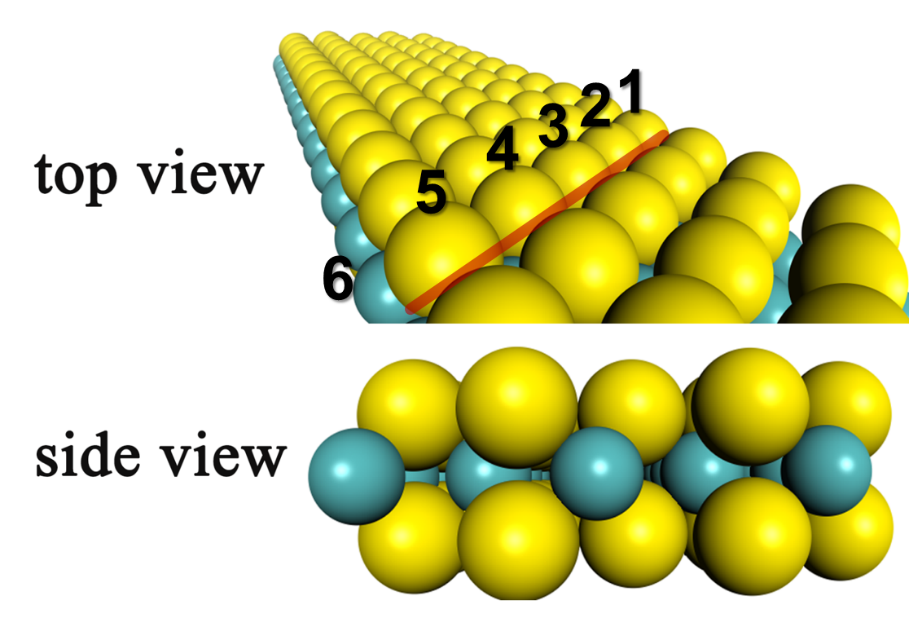
**Figure S4.** Top and side views of MoS_2_ nanostripe with 1nm width. The numbers from 1 to 6 represent six different adsorption sites for oxygen.

**Table S3**.The results of O2 adsorption on six different sites on MoS_2_ nanostripe

| Adsorption site | E_ads_ (ev) |
| --- | --- |
| 1 | 0.214 |
| 2 | 1.738 |
| 3 | 1.697 |
| 4 | 1.278 |
| 5 | 1.007 |
| 6 | -3.035 |

**
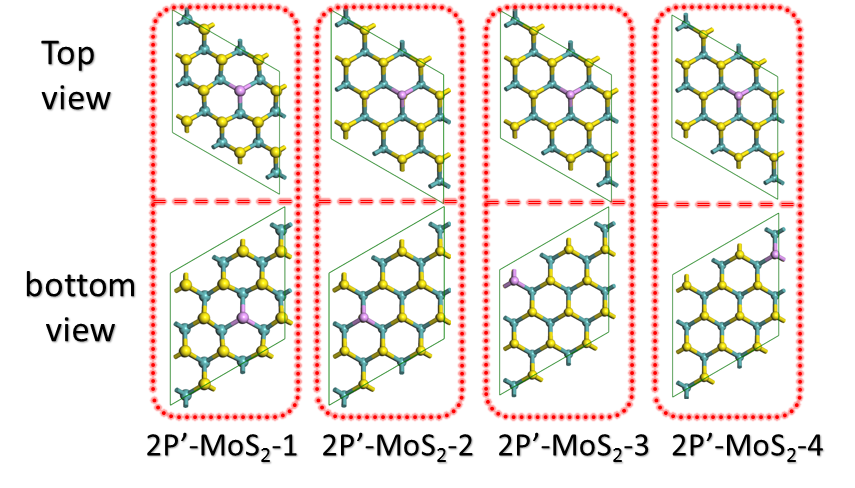
**

**Figure S5. Four different configurations of P doping on both sublayers.**

**Table S4**. The calculated bond energy, the height of P atom and formation energy for 2P’-MoS2.

|  | Mo-P bond energy (eV) | Relative height of P (Å) | Formation energy  (eV/atom) |
| --- | --- | --- | --- |
| 2P’-MoS2-1 | -- | -0.473 | -0.066 |
| 2P’-MoS2-2 | 0.090 (Mo-P) | -0.107 | -0.080 |
| 2P’-MoS2-3 | 0.184 (Mo-P) | -0.029 | -0.079 |
| 2P’-MoS2-4 | 0.180 (Mo-P) | -0.106 | -0.078 |

**Figure S6.** The condition that P present on the edge site.**
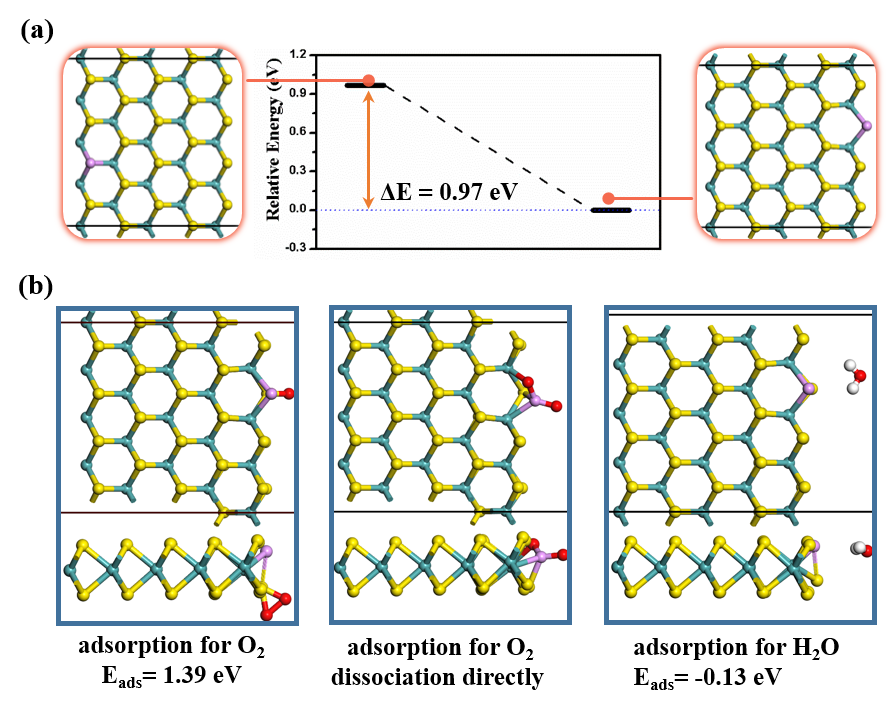
** (a) The relative energy difference between two edge sites that P could be placed. (b) The adsorption configuration and energy for O_2_ and H_2_O on MoS_2_ with P doping on edge site.

**Table S5.** Geometrical coordinates (Å) of three different types of 2P-doping MoS_2_ monolayers and the optimized structures of intermediates during ORR on the 2P-MoS_2_(1) monolayer.

**2P-MoS_2_ (1)**

**Atoms X Y Z**

P 0.918432 4.832732 14.925542

P 3.769518 6.478872 14.925542

Mo -3.740017 9.709176 13.387036

Mo 1.865802 3.209905 13.402844

Mo 10.271448 1.604176 13.396950

Mo 7.469491 0.016902 13.404987

Mo 7.484074 3.237299 13.387036

Mo 4.670956 1.625255 13.389447

Mo -0.940753 4.846137 13.393467

Mo -0.943725 8.111509 13.404987

Mo -3.719378 6.478677 13.396950

Mo 4.701241 4.847011 13.402844

Mo 4.687500 8.095675 13.393467

Mo 1.862157 6.490237 13.440624

S 3.726557 0.017874 14.964930

S 9.341855 3.220882 14.963858

S 0.920451 1.591159 14.968412

S 3.738111 0.005051 11.827363

S 9.351390 3.220882 11.823879

S 0.918488 1.604855 11.823344

S 3.727454 9.709954 14.970824

S 3.741644 3.234870 14.968947

S 6.535244 1.639535 14.964930

S 3.720893 9.711994 11.829239

S 3.738503 3.240310 11.829507

S 6.540628 1.623021 11.827363

S -4.654635 8.091984 14.963858

S -1.858792 6.484797 14.970824

S 0.941538 4.851383 11.842635

S -4.659459 8.083825 11.823879

S -1.857278 6.491500 11.829239

S 6.575849 4.856240 14.968412

S 0.912655 8.134823 14.971359

S 6.564912 4.864885 11.823344

S 0.927685 8.108789 11.840224

S 3.741812 6.468186 11.842635

**2P-MoS_2_ (2)**

**Atoms X Y Z**

P 0.910957 4.857186 14.925775

P 6.519086 4.857038 14.925395

Mo -3.790738 9.713994 13.393282

Mo 1.828668 3.234263 13.392825

Mo 10.226960 1.604832 13.400757

Mo 7.425896 0.000043 13.393379

Mo 7.436992 3.234190 13.392654

Mo 4.618777 1.604854 13.400888

Mo -0.964562 4.857114 13.400704

Mo -0.989818 8.109208 13.400672

Mo 7.436984 6.479772 13.392642

Mo 4.644039 4.856957 13.400784

Mo 4.618598 8.109162 13.400931

Mo 1.828561 6.479774 13.392595

S 3.675425 0.000056 14.947081

S 9.309399 3.232716 14.928429

S 0.880330 1.615024 14.930443

S 3.683094 0.000298 11.857058

S 9.317230 3.228584 11.865706

S 0.874571 1.612635 11.865931

S 3.675434 9.713879 14.947167

S 3.701124 3.232421 14.928490

S 6.489124 1.614908 14.930198

S 3.683219 9.713932 11.856879

S 3.708763 3.228742 11.865680

S 6.482971 1.612730 11.865860

S 6.488997 8.098922 14.930264

S -1.907651 6.481469 14.928103

S 0.898124 4.856902 11.854176

S 6.482733 8.101372 11.865747

S -1.899822 6.485446 11.865622

S 0.880521 8.099075 14.930365

S 3.700910 6.481375 14.928325

S 6.506145 4.857024 11.854167

S 0.874498 8.101219 11.865694

S 3.708743 6.485296 11.865796

**2P-MoS_2_ (3)**

**Atoms X Y Z**

P 5.608400 3.238011 15.096747

P 0.000000 6.476023 15.096747

Mo 0.945924 4.858211 13.579878

Mo 0.928104 8.104123 13.579878

Mo -1.874029 6.465735 13.579878

Mo 3.734372 3.248299 13.579878

Mo 6.554324 4.855823 13.579878

Mo 6.536505 1.609911 13.579878

Mo -1.856647 3.215807 13.599766

Mo 3.751753 6.498226 13.599766

Mo 3.713295 0.000000 13.599766

Mo 0.933067 1.616120 13.580889

Mo 9.350665 0.000000 13.580889

Mo -4.675333 8.097913 13.580889

S 2.789816 1.628052 15.125012

S 8.411957 1.602025 15.125012

S 5.623426 6.483956 15.125012

S 0.015026 3.230078 15.125012

S -2.818584 8.085981 15.125012

S 2.803558 8.112008 15.125012

S 2.799311 1.610659 12.052648

S 8.422272 1.618944 12.052648

S 5.603617 6.484430 12.052648

S -0.004783 3.229604 12.052648

S -2.809089 8.103373 12.052648

S 2.813873 8.095089 12.052648

S -2.794191 4.839681 15.120288

S 2.814208 4.874352 15.120288

S 5.588383 0.000000 15.120288

S -2.784084 4.822174 12.053216

S 2.824316 4.891859 12.053216

S 5.568167 0.000000 12.053216

S 5.608400 3.238011 12.043859

S 0.000000 6.476023 12.043859

S 0.000000 0.000000 15.118434

S 0.000000 0.000000 12.055369

**2O-2P-MoS_2_ (1)\*2O**

**Atoms X Y Z**

O 0.935088 4.729860 16.455473

O 3.847082 6.411359 16.455742

P 0.919441 4.827875 14.953406

P 3.771200 6.474597 14.953406

Mo -3.695262 9.645841 13.380873

Mo 1.967595 3.186106 13.324606

Mo -0.898690 1.559879 13.380606

Mo 1.912857 9.637001 13.393734

Mo 7.513910 3.164832 13.381142

Mo 4.712346 1.549680 13.386767

Mo -0.927461 4.766579 13.364529

Mo -0.891399 8.017963 13.393734

Mo 7.509254 6.414468 13.380873

Mo 4.666637 4.743751 13.325410

Mo 4.745996 8.042929 13.364798

Mo 1.932318 6.364052 13.342826

S -1.827833 9.640498 14.960641

S 9.377693 3.166192 14.963858

S 0.998295 1.593879 14.922862

S -1.819421 9.634864 11.821468

S 9.369000 3.139867 11.794943

S 0.977881 1.541034 11.798962

S 3.795541 9.649727 14.955283

S 3.767274 3.184649 14.966001

S 6.583757 1.545211 14.960375

S 3.766377 9.658275 11.810215

S 3.801934 3.126364 11.745374

S 6.584542 1.535109 11.821736

S -4.628613 8.032535 14.963858

S -1.843481 6.393388 14.955283

S 0.956625 4.771436 11.785832

S -4.601524 8.026026 11.795211

S -1.836919 6.423016 11.810483

S 6.531206 4.788047 14.923130

S 0.994481 7.989210 14.950996

S 6.586112 4.779596 11.800034

S 0.970590 8.030203 11.808071

S 3.800532 6.412914 11.786636

**H_2_O+2O-2P-MoS_2_ (1)\*2O+*H_2_O**

**Atoms X Y Z**

H 3.593302 4.988156 17.535000

H 2.335057 4.148767 17.614042

O 0.845074 4.721992 16.349905

O 3.836706 6.474015 16.345619

O 3.220624 4.223565 18.024525

P 0.870087 4.820492 14.843553

P 3.739737 6.486157 14.838193

Mo -3.740915 9.655944 13.276110

Mo 1.918129 3.191740 13.228683

Mo 10.271896 1.569205 13.275842

Mo 1.866307 9.646521 13.290042

Mo 7.467809 3.174352 13.278253

Mo 4.667311 1.558131 13.282271

Mo -0.977208 4.780276 13.258426

Mo -0.937220 8.027775 13.290578

Mo 7.464275 6.424181 13.276377

Mo 4.626257 4.754631 13.230291

Mo 4.699503 8.058568 13.254674

Mo 1.883525 6.376680 13.245564

S -1.874496 9.649240 14.855877

S 9.332321 3.177752 14.857754

S 0.949895 1.598639 14.822386

S -1.864400 9.644773 11.717508

S 9.321441 3.150553 11.690715

S 0.931443 1.552691 11.697412

S 3.749496 9.659149 14.849447

S 3.724539 3.189700 14.860165

S 6.538609 1.553857 14.856682

S 3.717696 9.671680 11.702771

S 3.755553 3.138507 11.646772

S 6.538385 1.544531 11.718043

S 6.540292 8.043220 14.855877

S -1.889358 6.403491 14.851590

S 0.910972 4.780956 11.687767

S 6.571418 8.037100 11.688839

S -1.884198 6.435936 11.704647

S 6.490714 4.798150 14.823189

S 0.947539 7.999798 14.849447

S 6.539170 4.789990 11.699824

S 0.926059 8.037197 11.708666

S 3.752412 6.421656 11.688035

**OH+O-2P-MoS_2_ (1)\*O+*2OH**

**Atoms X Y Z**

H 2.771559 5.926143 16.686438

H 2.666850 3.705224 16.575510

O 1.370749 4.726557 16.392241

O 3.612595 6.414759 16.436985

O 3.543275 3.181540 16.412336

P 1.038731 4.784842 14.874901

P 3.733624 6.421365 14.826941

Mo -3.694870 9.633893 13.403381

Mo 1.885432 3.120730 13.318711

Mo 10.309641 1.529280 13.412759

Mo 1.910501 9.640304 13.410614

Mo 7.527369 3.161529 13.429906

Mo 4.730405 1.507715 13.423475

Mo -0.912711 4.776585 13.423208

Mo -0.895774 8.024958 13.417314

Mo -3.676138 6.403394 13.430710

Mo 4.761980 4.782802 13.378462

Mo 4.737360 8.056722 13.398825

Mo 1.890424 6.426707 13.308798

S -1.834788 9.624372 14.993598

S -1.817851 3.154438 15.004315

S 0.972160 1.552497 14.955551

S -1.825927 9.632728 11.840224

S 9.379432 3.146084 11.851478

S 0.929480 1.484013 11.798426

S 3.776696 9.630687 14.990383

S 3.725435 3.180180 14.805505

S 6.597778 1.529280 14.997349

S 3.763797 9.648950 11.829774

S 3.817413 3.135884 11.817182

S 6.595703 1.549971 11.851746

S -4.626145 8.030397 14.991454

S -1.807699 6.400771 15.005120

S 0.930546 4.763567 11.796818

S -4.597598 8.005043 11.838616

S -1.824917 6.412331 11.852014

S 6.591328 4.789990 14.994669

S 0.989154 7.993192 14.959301

S 6.646739 4.787853 11.837544

S 0.934640 8.082951 11.797890

S 3.825433 6.414273 11.813430

**H_2_O+O-2P-MoS_2_ (1)\*O+H_2_O**

**Atoms X Y Z**

H 3.899352 5.721080 22.189100

H 2.672403 6.469352 21.673586

O 0.957185 4.747928 16.435377

O 3.645628 6.525013 21.699039

P 0.985508 4.746665 14.925542

P 3.781744 6.379012 14.931168

Mo -3.680344 9.624276 13.387036

Mo 1.950321 3.112668 13.368012

Mo -0.879061 1.535206 13.388376

Mo 1.920540 9.640401 13.391859

Mo 7.541167 3.153370 13.383018

Mo 4.733714 1.535206 13.384089

Mo -0.908505 4.768133 13.362922

Mo -0.876593 8.012717 13.383553

Mo -3.670810 6.393194 13.392931

Mo 4.731190 4.754340 13.373103

Mo 4.735789 8.013009 13.390251

Mo 1.942582 6.424765 13.369083

S -1.808148 9.627967 14.954746

S -1.808709 3.161335 14.957963

S 0.995827 1.531709 14.957695

S -1.813084 9.631853 11.820129

S 9.397379 3.131707 11.801909

S 0.979114 1.508007 11.806731

S 3.792512 9.636127 14.960375

S 3.790886 3.147055 14.960641

S 6.597610 1.544920 14.959035

S 3.786399 9.632144 11.822272

S 3.810235 3.142879 11.792799

S 6.600918 1.538800 11.816378

S -4.609039 8.006598 14.961713

S -1.797829 6.384840 14.958230

S 0.998351 4.760945 11.841832

S -4.606235 8.002906 11.819593

S -1.816336 6.410873 11.804588

S 6.599011 4.772602 14.950728

S 0.990444 8.017574 14.955283

S 6.617743 4.778236 11.816378

S 0.985284 8.043802 11.802176

S 3.817637 6.394554 11.801641

**2OH-2P-MoS_2_ (1)\2*OH**

**Atoms X Y Z**

H 4.366532 5.986856 17.010643

H 1.795024 5.258790 16.899715

O 0.969804 4.835258 16.557558

O 3.569746 6.327916 16.547377

P 0.979171 4.788144 14.936259

P 3.746186 6.374932 14.919915

Mo -3.711751 9.626607 13.516183

Mo 1.924747 3.113639 13.482959

Mo 10.303472 1.535497 13.520202

Mo 1.890536 9.640498 13.512700

Mo 7.513517 3.154438 13.518862

Mo 4.701634 1.536760 13.511360

Mo -0.947876 4.769494 13.506805

Mo -0.909850 8.024278 13.511360

Mo 7.511442 6.400771 13.519933

Mo 4.728442 4.723935 13.482690

Mo 4.730461 8.049048 13.498499

Mo 1.869112 6.439141 13.452949

S -1.841742 9.634476 15.087645

S 9.374777 3.154729 15.098899

S 0.967281 1.540160 15.082018

S -1.847968 9.635933 11.944452

S 9.365130 3.133747 11.938826

S 0.943445 1.506841 11.934271

S 3.763068 9.629619 15.088984

S 3.765872 3.151718 15.081215

S 6.568838 1.539189 15.087645

S 3.755104 9.649824 11.937219

S 3.783090 3.126170 11.905869

S 6.571587 1.546668 11.947132

S 6.571587 8.013689 15.096219

S -1.837144 6.398343 15.089520

S 0.943052 4.752008 11.939630

S -4.618405 8.011552 11.935075

S -1.848472 6.411359 11.940434

S 6.569456 4.776196 15.086038

S 0.960383 8.015826 15.075855

S 6.610901 4.775321 11.934539

S 0.929704 8.072847 11.906672

S 3.798962 6.395429 11.940434
